# Predicting Power Output of Upper Body using the OMNI-RES Scale

**DOI:** 10.2478/hukin-2014-0122

**Published:** 2014-12-30

**Authors:** Iker J. Bautista, Ignacio J. Chirosa, Ignacio Martín Tamayo, Andrés González, Joseph E. Robinson, Luis J. Chirosa, Robert J. Robertson

**Affiliations:** 1 CTS 642 Research Group Dept. of Physical Activity and Sport. University of Granada (Spain).; 2 Department of Methodology of Behavioural Sciences. University of Granada (Spain).; 3 Department of Health and Physical Activity, Center for Exercise and Health-Fitness Research, University of Pittsburgh, Pittsburgh, Pennsylvania.

**Keywords:** resistance training, upper body, bench press, perceived exertion, mean velocity prediction

## Abstract

The main aim of this study was to determine the optimal training zone for maximum power output. This was to be achieved through estimating mean bar velocity of the concentric phase of a bench press using a prediction equation. The values for the prediction equation would be obtained using OMNI–RES scale values of different loads of the bench press exercise. Sixty males (age 23.61 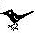 2.81 year; body height 176.29 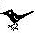
6.73 cm; body mass 73.28 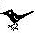 4.75 kg) voluntarily participated in the study and were tested using an incremental protocol on a Smith machine to determine one repetition maximum (1RM) in the bench press exercise. A linear regression analysis produced a strong correlation (r = −0.94) between rating of perceived exertion (RPE) and mean bar velocity (Vel_mean_). The Pearson correlation analysis between real power output (Pot_Real_) and estimated power (Pot_Est_) showed a strong correlation coefficient of r = 0.77, significant at a level of p = 0.01. Therefore, the OMNI–RES scale can be used to predict Vel_mean_ in the bench press exercise to control the intensity of the exercise. The positive relationship between Pot_Real_ and Pot_Est_ allowed for the identification of a maximum power-training zone.

## Introduction

Control of intensity is one of the fundamental pillars of strength and conditioning and sport science practice. Therefore, researchers and coaches assess training intensity through identification of maximum dynamic strength (1RM–one repetition maximum) and train athletes within their corresponding percentages (Bird et al., 2005; Fleck, 1999). Different training objectives can be reached by working within certain percentages of 1RM. For example, to develop maximum strength, it is necessary to train using loads > 80% of 1RM. To develop the ability to produce force in relation to velocity (i.e. power) it is necessary to train using loads between 40% – 80% of 1RM, and to move the load as quickly as possible (Baker et al., 2001; Cronin et al., 2001).

Other ways to control the intensity of strength exercises include the choice and order of exercises, the type of sessions, load, rest periods between sets, the velocity of the load displacement or repetitions to fatigue (Bird et al., 2005; De Salles et al., 2010; De Salles et al., 2009; Fleck and Kraemer, 1997; Kamawori and Newton, 2006; Tiggerman et al., 2010). With the development of new technologies, strength training has evolved significantly, especially from a quantitative point of view (Bosquet et al., 2010; Harris et al., 2010; Rontu et al., 2010). Devices such as linear position transducers (LPT) are able to quantify, indirectly, variables such as force production, power output, displacement and velocity (Harris et al., 2010). The information provided by these devices is very useful for planning and periodization of macro or micro cycles, individual and group training sessions and the number of repetitions performed of a particular exercise, i.e. bench press.

Typically, controlling velocity has been a commonly used means of prescribing specific strength training (Bosco et al., 1995; Farthing and Chilibeck, 2003; Haff et al., 2001; Kamawori and Haff, 2004; Pereira and Gomes, 2003). By controlling execution velocity the neural effects of motor unit recruitment can be targeted in accordance with the size principle (Cormie et al., 2011; Day et al., 2004; Jandacka and Vaverka, 2009). Moras et al. (2009) proposed an original method for controlling the velocity of bar displacement during bench press with the use of a metronome. The low standard error of measurement and coefficients of variance associated with the use of this device have shown it to be a valid and efficient way to estimate the mean velocity of the bar during a bench press.

Also, training while taking into account the athletes perceptions of exertion has become more of a common practice in recent years (Belleza et al., 2009; Day et al., 2004; Pfeiffer et al., 2002). In the last decade scales have been designed to measure perceived exertion in strength training. This increase in popularity is due to the need to know the subjective (i.e. internal) perceptions of athletes in relation to the external stimulus applied. According to Robertson et al. (2003), the perception of exertion can be defined as: “the subjective intensity of the effort, strain discomfort and/or fatigue experienced while performing an exercise.” The original scale of perceived exertion was proposed by Borg (with values between 6 and 20). This format was based on the strong correlation between rating of perceived exertion (RPE) and some physiological variables as a lactate level, heart rate, respiration rate, ventilation threshold and oxygen uptake (Coutts et al., 2009; Foster et al., 2001; Nakamura et al., 2010). The applicability of such scales was demonstrated by Suzuki et al. (2006), who predicted 400 m sprint performance using a mathematical model based on subjective scale scores (pain scale or “CP scale” and total quality of recovery scale “TQR scale”) with the Borg CR10 RPE scale as a control. The results illustrated a strong correlation (R2 = 0.83) between predicted and actual performance. The authors concluded that subjective scales of effort could effectively predict performance, allowing for the optimal manipulation of training variables to achieve peak performance.

However, the physiological variables listed above have little relation to the demands of strength training hence the limited use of the Borg RPE (6 – 20) scale within strength training settings. However, there is an eleven-point metric known as the OMNI–RES scale, which can be used to evaluate different intensities for both upper and lower body resistance exercises (Duncan et al., 2006; Suzuki et al., 2006; Robertson et al., 2003). Like Suzuki et al. (2006), Nacleiro et al. (2011) conducted a study to measure RPE, but with a focus on strength training. The authors analysed whether the OMNI–RES scale could be used to control the intensity of upper body strength training. A relation between RPE, external load and mechanical power in the bench press exercise was established. The main conclusion of the investigation was that the OMNI–RES scale can be used to control the intensity of strength training exercises such as the bench press.

In other research, Duncan et al. (2006) studied the relation between RPE during dynamic muscle activity in the leg extension exercise with electromyography at 30%, 60% and 90% of the 1RM. Both muscle activity and the values of the OMNI–RES scale increased with exercise intensity. The authors concluded that the regulation of resistance exercise intensity using this scale had valid practical applications, but suggested the need for coaches to differentiate between overall RPE and active muscle RPE, as the former was significantly lower than the latter.

Therefore, it is clear that researchers and coaches are interested in analysing the relation between RPE and intensity of upper and lower body strength training. To our knowledge, no study has analysed the relation between mean velocity (Vel_mean_) of the bar during the concentric phase of the bench press and RPE. Thus, the aim of this study was threefold, with the main aim (a) to determine the optimal training zone for maximum power output. This was to be achieved through (b) estimating mean bar velocity of the concentric phase of a bench press using a prediction equation. The values for the prediction equation would be (c) obtained using OMNI–RES scale values of different loads of the bench press exercise.

## Material and Methods

### Subjects

Sixty healthy males volunteered to participate in the study. The mean ± SD age, body height and body mass were 23.61 ± 2.81 years, 176.29 ± 6.73 cm and 73.28 ± 4.75 kg, respectively. All the subjects were students at the Faculty of Sport Sciences. The inclusion criteria for this study were: (a) a minimum of two years of experience with resistance training up to the time of study, (b) a prohibition on taking medications or dietary supplements (i.e. creatine) and (c) an absence of injury that could interfere with the execution of the exercise. Prior to data collection, all subjects were informed of the risks involved in the study and gave written informed consent to participate. The study was approved by the University of Granada Ethics Committee.

### Procedures

The experimental protocol was conducted in the Performance Control Laboratory. Prior to the evaluation sessions, the subjects’ height and body mass were measured, and the handgrip for the bench press exercise was standardised. In order to standardise the handgrip, subjects lay horizontally in a supine position with the elbow joint flexed at a 90^°^ angle. The bar was positioned 5 cm above the jugular notch. After taking anthropometric measurements and standardising the bench press exercise, the subjects were instructed how to use the OMNI-RES scale, according to the procedures explained by Robertson et al. (2003).

In the first evaluation session, subjects performed a standardised 5-min warm-up at 75 W on a cycle ergometer and two sets of 15 repetitions on bench press with a 20 kg load on the Smith machine (Gervasport, Madrid, Spain). Afterwards, subjects completed the incremental protocol until reaching 1RM. The Smith machine and the bar used were calibrated to avoid any influence on test results. The incremental protocol consisted of progressive increases of 10 kg loads (for mean bar velocities greater than 0.5 m · s^−1^) and increases of 5 kg loads (for mean bar velocities lower than 0.5 m · s^−1^). The initial load was 20 kg and loads were increased as described until the subject could only lift the bar once, the load at this point was designated the 1RM. All subjects performed 2–4 repetitions of all loads, except for the 1RM.

To avoid the affect of neural fatigue, length of the rest periods after each set was determined by mean bar velocity. For mean bar velocities > 0.5 m · s^−1^ the rest period was 3 min and for mean bar velocities < 0.5 m · s^−1^ the rest period was 5 min. The descent phase of the bar was controlled by verbal instructions from the researcher as follows; “down” (two seconds), “chest” (one second), “go”. To avoid the rebound effect, the “lift” signal was randomised.

Immediately after finishing the set, the subject gave his RPE using the OMNI-RES scale. The ratings given by each subject were collected for each load of the incremental protocol. In order to control velocity and power output (Pot_Real_) of the concentric phase of each repetition, a linear position transducer was attached to the bar (T– Force System, Ergotech, Murcia, Spain). The estimated power output (Pot_Est_) was then obtained through the following equation:

(equation 1) $${\mathrm{Pot}}_{\mathrm{Est}}=F\cdot \;{\mathrm{Vel}}_{\mathrm{Est}}$$

where F is the mass (load) multiplied by gravity (9.81 m · s^−2^); and Vel_Est_ is established using the OMNI–RES scale values (see equation 2).

### Statistical Analysis

All data were expressed as mean ± SD for the mean velocity variables and OMNI–RES scale responses. Normality distributions of variables was assessed using the Kolmogorov–Smirnov test. Pearson correlations were analysed to establish a relation between Vel_mean_ of the bar and OMNI-RES scale. Finally, linear regression analyses were conducted to create a model for predicting bar velocities using RPE derived from the OMNI–RES scale. Pearson correlation analysis was performed to establish a relation between Pot_Est_ and Pot_Real_. To solve the problem of non-independence of data when performing a linear correlation analysis, subjects were required to participate in one load category only. This ensured each subject in the sample was used only once. All analyses were conducted using SPSS v 20.0 for Mac (Chicago, IL). The alpha level was set at p < 0.05.

## Results

The Vel_mean_ of the bar and RPE (OMNI– RES scale) were recorded for each load of the incremental protocol. Those data are listed as mean ± SD in Table 1.

Simple linear regression analyses were performed between Vel_mean_ of the bar (predicted variable) and RPE (predictor). The regression coefficient for the 60 scores was r = −0.939, which explained 88.1 % of the variability between Vel_mean_ and RPE obtained from the OMNI-RES. The regression analyses produced the following model to predict mean bar velocity:

(equation 2) VelEst=(−0.1047⋅value OMNI−RES)+(1.2276)

The 95% confidence intervals for the equation were Vel_Est_ = (−0.095 · value OMNI-RES) + (1.286) for the upper limit and Vel_Est_ = (−0.115 · value OMNI-RES) + (1.169) for the lower limit.

**Figure 2:**
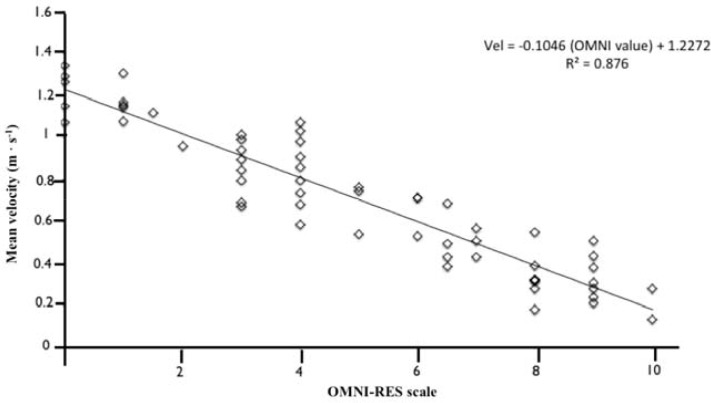
Relationship between mean velocity (y-axis) and the OMNI–RES scale scores (x-axis). The black points represent average real power output.

Figure 2 illustrates the mean values of the OMNI–RES scale and the Vel mean of execution in a ‘power curve’ through a spectrum of loads from 20 to 70 kg.

To calculate real power (Pot_Real_) data from the LPT was used. Estimated power (Pot_Est_) values were calculated using equation 1, with velocity estimated (Vel_Est_) using equation 2. Combining both equations gives an overall power output value as illustrated in the following equation:

(equation 3) PotEst=(m⋅g)⋅[(−0.1047⋅value OMNI−RES)+(1.2276)]

The Pearson correlation analysis between Pot_Real_ and Pot_Est_ showed a strong correlation coefficient of r = 0.765, significant at a level of p = 0.01.

The velocity for each zone can be differentiated by using the calculations from equation 2, and therefore using RPE (OMNI–RES values) to predict Vel_Est_ (m · s^−1^).

## Discussion

The main aim of this study was to determine the optimal training zone for maximum power output, using the OMNI–RES scale. The analyses of this study show that it is possible to predict power output using the standardised formula for power (P = F · V), where, in this instance, force (F) is derived from the mass of the bar and weight plates multiplied by the force of gravity (9.81 m · s^−2^) and velocity (V) is established from the OMNI– RES scale values for the corresponding load.

Different authors (Nacleiro et al., 2011; Robertson et al., 2008; Sweet et al., 2004) have correlated the intensity of strength exercises (mean percentages of 1RM) with RPE from the OMNI–RES Scale. Nacleiro et al. (2011) established seven percentage ranges (30–40%, 40– 50%, 50–60%, 60–70%, 70–80%, 80–90%, >90%) of 1RM with an approximated RPE (OMNI-RES) following the first three repetitions (2.2 ± 1.2, 2.3 ± 1.2, 2.4 ± 1.6, 3.2 ± 2.2, 6.8 ± 1.0, 7.7 ± 1.1, 8.6 ± 0.2, respectively). Gearhart et al. (2009) showed that the use of the OMNI–RES could control the intensity of different strength exercises (i.e. bench press, leg extension, arm extension etc.) over a 12– week training period in men and women. The results demonstrated a significant difference (p < 0.05) in the RPE values for the first and last training period for all exercises used. In conclusion, Gearhart et al. (2009) stated that the OMNI–RES was a valid tool for controlling performance based on perception of exertion. These findings agree with those in the present study that also proved RPE derived from the OMNI–RES to be a viable tool for controlling resistance exercise performance. In this instance, the mean bar velocity of the bench press and RPE were measured for each load. The findings indicated that by using this type of scale it is possible to quantify the intensity of strength training by measuring mean velocity of the execution of the exercise. Therefore, using equation 1 to predict velocity and equation 2 to predict power, the power output can be estimated (equation 3) during the execution of the exercise. It is then possible to calculate the optimal training zone for maximum power (see Figure 2).

In order to quantify the intensity of strength training, Robertson et al. (2008) established various equations to predict 1RM of the knee extension (KE) and bicep curl (BC) in children aged 10–14 years. The models proposed by the authors demonstrated a strong positive relation (*R*^2^ = 0.76–0.79) between 1RM scores for both KE and BC. However, Wood et al. (2004) demonstrated that subjective perception of effort in the KE exercise increased as the training session progressed and the number of sets increased. They found that significant RPE differences increased not only from one set to the next, but as the number of repetitions in each set increased. In the present study, the Vel_mean_ of the bar for each load was correlated with the RPE given by each subject immediately after performing each set. The Pearson correlation analysis revealed strong negative correlation coefficients between all pairs of scores for loads 20 to 70 kg and the RPE (Table 2). Unlike the study of Nacleiro et al. (2011), in our research RPE (OMNI-RES) successfully discriminated between the mean velocities of the loads lifted by the participants.

Other studies such as that of Pincivero et al. (2001) have used the Borg’s CR–10 RPE scale to analyse exertion responses to maximum voluntary contraction (MVC) intensity in men and women, by means of leg extension on an isokinetic dynamometer. The results showed that RPE underestimated MVC in sub-maximal loads of the leg extension. However, contrary to Pincivero et al. (2001), findings of the present study showed that RPE formed the OMNI-RES effectively distinguished between mean bar velocity for each load lifted as subjects were able to perceive different exercise intensities (i.e. % 1RM) within 3–4 repetitions, that is, when a subject perceived a high intensity in the RPE scale the execution velocity was low and vice versa. This demonstrates that scales of perceived exertion can be used effectively to control the intensity of strength training. Mean bar velocity is a good indicator of the specific aspect of strength that is being trained; i.e. maximum strength or the rate of force development (González–Badillo and Sánchez–Medina, 2010; Haff et al., 2001; Kamawori and Haff, 2004; Pereira and Gomes, 2003). For example, in the bench press, mean bar velocities of 0.15 to 0.30 m · s^−1^ (> 80% 1RM) improves maximum strength and velocities of 0.5 to 0.7 m · s^−1^ (60 – 80% 1RM) are associated with enhanced maximal power (Figure 2 and Table 3). The findings of the present study together with those of previous research (Day et al., 2004; Lagally et al., 2004; Nacleiro et al., 2011) have demonstrated the accuracy of RPE scales in effectively controlling the intensity of strength exercises. In case of the present study, this was achieved by predicting the execution velocity of the exercise using a RPE based model from which the power output can be predicted.

The positive relationship between real power and estimated power allowed for the identification of a maximum power–training zone. The findings of this study therefore suggest that using a specific prediction formula, bar velocity can be calculated and the intensity of training sessions can be determined without the use of LPT.

## Practical application

Practitioners can benefit from using metrics such as the OMNI-RES, which is an effective, cost efficient and simple tool that provides instant feedback on exercise intensity.

As said the OMNI-RES allows intensity control during the training session and provides invaluable information that the desired training outcomes are achieved. Another advantage of using subjective scales of effort is the information provided about individual perception. This can help determine a progressive increase of the training load via the athletes’ OMNI-RES score. For example, if lifting 40 kg is perceived as a score of 6 and following a training intervention, 40 kg is perceived as a score of 4, the training load can increase as power output has improved. This means that athletes are required to listen to the sensations produced after the execution of any exercise, and therefore develop understanding of perceived effort and actual capabilities.

## Figures and Tables

**Figure 1:**
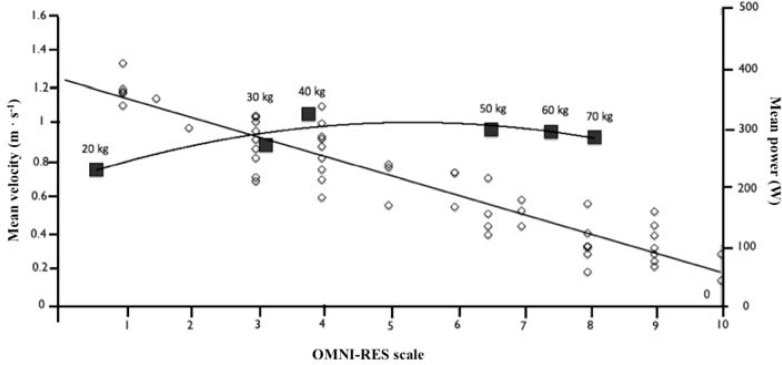
Regression analysis of the Vel_mean_ of the bar and OMNI–RES scale scores.

**Table 1:** Mean ± SD of Vel_mean_ of the bar and OMNI–RES score for each load

**Load (kg)**	**20**	**30**	**40**	**50**	**60**	**70**
**N**	10	10	10	10	10	10

**Vel_mean_ (m·s^−1^)**	1.17	0.92	0.80	0.55	0.44	0.35
***SD***	0.12	0.11	0.19	0.26	0.18	0.12

**OMNI–RES**	0.60	3.80	4.15	6.70	7.55	8.05
***SD***	0.70	0.79	1.20	2.31	1.28	1.26

**Table 2:** Pearson correlation analysis between mean bar velocity and OMNI–RES scale scores for each load.

**Load (kg)**	**N**	**Pearson Correlation (*r*)**
**20**	10	−0.59
**30**	10	−0.67^*^
**40**	10	−0.90^**^
50	10	−0.89^**^
**60**	10	−0.71^**^
**70**	10	−0.73^*^

**Table 3:** ONMI-RES scale values and corresponding average velocity calculated by the prediction formula.

	**OMNI-RES Scale Score**	**Mean Velocity (m · s^−1^)**
**Maximum Power Zone**	4–6	0.76 – 0.54
**Maximum Strength Zone**	7–9	0.44 – 0.22
